# Impacts of Emergency Remote Teaching on College Students Amid COVID-19 in the UAE

**DOI:** 10.3390/ijerph19052979

**Published:** 2022-03-03

**Authors:** Alaa El-Sakran, Reem Salman, Ayman Alzaatreh

**Affiliations:** Department of Mathematics and Statistics, American University of Sharjah, Sharjah P.O. Box 26666, United Arab Emirates; g00042916@alumni.aus.edu (A.E.-S.); g00071008@alumni.aus.edu (R.S.)

**Keywords:** COVID-19, emergency remote teaching, confirmatory factor analysis, structural equation modeling, quality of courses, academic performance, psychological distress, readiness for future work or studies

## Abstract

With the aim of appraising the impact of Emergency Remote Teaching (ERT) amidst the COVID-19 pandemic on college students, an online survey was conducted in December 2020 on a total of 588 undergraduate students at the American University of Sharjah in the United Arab Emirates. The purpose of the study was to probe into the perceptions of college students regarding their learning process and its influence on their mental health with the abrupt transition from face-to-face classes to ERT in the Spring 2020 semester. A comprehensive analysis was performed using structural equation modeling and other statistical techniques to reveal crucial results associated with the factors that have an effect on the students’ psychological distress, such as quality of courses, academic performance, and readiness for future work or studies. Findings suggest that the students’ perceived quality of courses under ERT has a significant impact on their academic performance and readiness for future work or studies. Moreover, they indicate that these factors serve as a vital mediating role in provoking psychological distress among the students. The study also shows that gender, previous history of anxiety/distress, education being at risk due to financial issues caused by COVID-19, and year of study significantly affect the students’ distress levels. In order to ensure and prioritize the well-being of college students during these turbulent times, new strategies are urgently needed to develop and enhance resilient ERT environments in higher education. The study concludes with limitations and suggestions for further research.

## 1. Introduction

Unforeseen calamities could turn people’s life upside down and make them feel helpless, distressed, and paralyzed. An example of this is the Coronavirus pandemic known as COVID-19. It started in the last quarter of the year 2019; it is a severe respiratory syndrome that has taken the whole world by storm. It began in Wuhan, China [1] and then spread to more than 200 countries [2]. On the 11th of March (2020), it was announced as a global pandemic by the World Health Organization [3]. Millions of people from various sectors as well as students were obligated by their national governments to stay safe by quarantining themselves through an entire or partial lockdown [4]. Consequently, public health, especially mental health, has become at risk [5]. A systematic review of the literature on mental health following the COVID-19 pandemic revealed relatively high rates of anxiety, depression, post-traumatic stress disorder (PSTD), psychological distress, and stress among the general population in eight countries [5]. In Italy, a study of mental health during the lockdown suggested that the pandemic is associated with great levels of psychological distress that could meet the threshold for clinical relevance [6]. These issues possess the potential for inducing long-term effects. Particularly, psychological distress effects play a crucial role in developing higher levels of PSTD symptomatology [7]. As such, there is a pressing need to improve mental healthcare in the immediate future, especially for those at particular risk for long-term consequences during the COVID-19 pandemic. This includes younger age individuals, females, and those with a previous history of stress and medical conditions [8]. Furthermore, the academic and relational changes ascribed to the COVID-19 lockdown were found to have significant impacts on the mental health of students in particular [9].

Due to the imposed lockdown, physical access to classrooms has been constrained and most educational institutions had to rapidly shift to online learning. According to the United Nations, nearly 1.6 billion students all over the world have been affected by the closure of educational institutions under COVID-19 [10]. Moreover, it is estimated that 80–85% of the student population has shifted to online learning in high-income countries, whereas less than 50% have made the shift in low-income countries [10]. In the United Arab Emirates (UAE), 82.86% of the total number of schools have opted for continued operation with online learning [11]. As the prominent alternative to the closure of educational institutions during this period, online learning has become a subject of particular interest to governments and researchers alike. Hence, the current researchers, because of the online learning environment induced by the COVID-19 pandemic, have decided to call it Emergency Remote Teaching (ERT) [12].

ERT is defined as “a temporary shift of instructional delivery to an alternate delivery mode due to crisis circumstances” [12]. It typically involves the utilization of remote teaching in place of face-to-face or blended courses. However, it should be emphasized that ERT differs from the standard online learning environment. Rather than creating high-quality online learning classes, the objective of ERT is to reliably establish temporary access to education in a prompt and accessible manner [12]. Once the crisis has passed, courses taught under ERT are expected to return to their original format [13]. In view of this, ERT faces a variety of obstacles and constraints that are distinct from the traditional online learning experience such as limited time for planning, access to support systems, or faculty training [13]. In fact, several challenges that both instructors and students encounter with ERT due to COVID-19 have been highlighted in the literature. Some of these difficulties are technology connectivity issues, unconducive physical environments, mental-health-related struggles, lack of teaching and learning resources [14]. Nevertheless, there are copious blessings associated with computer-mediated communication [15]. Some of these, for instance, are flexibility [16], self-pacing [17], and accessibility [18]. Consequently, the past decade has witnessed a growing interest in the utility of online learning by decision makers and practitioners worldwide [19].

Thus, the objective of this study is to probe the perceptions of college students in the UAE regarding the impact of the quality of courses during ERT on the students’ academic performance, psychological distress, and readiness for future work or studies. Each of these constructs is measured by some manifest variables in order to conduct a comprehensive study on the consequences of ERT. To date and to the best of the researchers’ knowledge, there has been no study that examined the effect of courses’ quality during ERT on the students’ readiness for future work or studies. Furthermore, we are not aware of any comprehensive analysis that has been conducted on the mediating role of academic performance, and readiness for future work or studies in examining the psychological distress of college students during the COVID-19 pandemic. Overall, this research could be of additional relevance considering the improvement of ERT during the COVID-19 pandemic and the effects of implementing ERT in the long run. In addition to drawing the attention of governments and policy makers to the mental health of college students during the pandemic, this study aims to provide suggestions that could potentially enhance the delivery of courses during the COVID-19 pandemic and any other similar circumstances.

## 2. Literature Review

Many researchers have addressed the importance of analyzing the impact of ERT on students, including those in higher education [20,21,22,23,24]. In a global survey conducted by the International Association of Universities (IAU), almost all respondents reported that COVID-19 has affected teaching and learning and that about two-thirds of institutions have shifted to ERT [25]. Moreover, 80% of higher education institutions believed that COVID-19 would have an impact on the enrollment numbers for the new academic year, particularly with respect to the international students’ mobility [25]. In turn, a decline in the proportion of international students could have a significant effect on the financing mechanism of higher education institutions for which international students incur higher tuition fees than domestic ones [26]. In the United States, a survey on college students’ experiences revealed that 13% of them have delayed graduation due to COVID-19; 40% have lost a job, internship, or job offer; and 29% expect to earn less at age 35 [27]. Therefore, as ERT becomes more widespread in the UAE, understanding the experiences of students and their response to ERT becomes especially important. In order to evaluate the impact of ERT, notably its effects on mental health and the well-being of college students, it is essential to recognize the many dimensions associated with ERT implementation.

### 2.1. Impact of ERT Dimensions

One of the main challenges related to understanding the impact of ERT on students comes with identifying its dimensions in a way that is reasonable to measure or evaluate. For the most part, the effectiveness of online learning systems cannot be evaluated using a single proxy construct or single-item scale [28]. The literature is rich on studies that discuss how standard online learning impacts students’ skills, participation, or satisfaction in comparison to traditional face-to-face learning [29,30,31,32]. Many researchers attempted to measure the impact of online learning through different conceptual models [33,34,35] or by investigating the main factors influencing online learning, relative to the COVID-19 pandemic [18,36,37]. Previous work pointed out the value of evaluating e-learning success from an Information Systems (IS) perspective [38,39,40,41,42]. One of the most popular IS Success models is the IS-Impact Measurement model [43]. This model consists of six IS success categories or dimensions: System Quality, Information Quality, Use, User Satisfaction, Individual Impact, and Organizational Impact. While the model is generally applied to institutions, the Individual Impact dimension can be used to asses the impact of e-learning on students [44]. An extension of the original model has combined the two individual and organizational impacts into a dimension termed “Net-Benefits” and added another dimension referred to as “Service Quality” [45]. The updated model has been used to evaluate the effectiveness of e-learning in [39].

A summary of recent literature on the constructs used to investigate the impact of ERT during the COVID-19 pandemic is presented in Table 1. According to Najmul and Yukun [46], an online focus group discussion (OFGD) was conducted with a sample of students in order to extract the dimensions of ERT, resulting in the creation of the scales of e-learning crack-up and fear of academic year loss. According to their work, there were several triggers of ERT such as course quality, availability of technical assistance, technological ease, and the probability of interaction between classmates. There was also an indication that the challenges of online learning could be split into some categories, such as technological, personal, and institutional challenges. Other works viewed the effectiveness of online learning in terms of knowledge gain [47,48] or academic performance [49]. In particular, student satisfaction was deemed one of the most crucial components in evaluating the quality of online learning [50,51,52]. Although these dimensions were commonly used to determine the success of standard online learning environments, recent studies identified their value in evaluating ERT during COVID-19 [53].

On the other hand, while several prior works focused on investigating the students’ attitudes towards ERT during COVID-19, very few had explored the effects of online learning and course quality under ERT in terms of technical-skills acquisition or readiness for future work and/or studies. Generally speaking, students tended to show a preference for face-to-face learning components over online learning in terms of gaining conceptual and methodical knowledge [59]. In the UAE, some of the main challenges for the applied science teachers during the COVID-19 pandemic included the absence of hands-on activities and conducting experiments in wet labs [60]. While there is evidence of the educational advantages of virtual laboratories and other online learning tools [61,62], it should be noted that the sudden shift to ERT has not made it possible for teachers and faculty to prepare a well-planned online curriculum that supports the transition.

### 2.2. Impact of ERT on Mental Health

In addition to its impact on education, the COVID-19 pandemic was also observed to have a significant impact on the public’s mental health. In the UAE, there were notable increases in the levels of anxiety and depression among adults in contrast to previous (pre-pandemic) national studies [63]. Some of the variables found to be significantly associated with both depression and anxiety included younger age, being female, having a history of mental health problems, self or loved ones testing positive for COVID-19, and having high levels of COVID-related anxiety and economic threat [63].

In general, some demographic variables were associated with developing psychological distress symptoms among students during COVID-19 [5]. Findings suggested that university students suffered from psychological distress due to ineffective online learning systems and fear of academic year loss [46]. In China, 24.9% from a sample of 7143 college students experienced anxiety during the pandemic [55]. Moreover, results showed that there was a significant positive correlation between the mental health of college students and the economic impacts of the pandemic. In addition, social support and presence of anxiety were found to be negatively correlated. Accordingly, students who lived with their parents had less anxiety levels as they were more likely to receive social support [55]. In the UAE, a cross-sectional study conducted on 433 college students from the University of Sharjah revealed that 51% of respondents were in psychological distress [64]. Additionally, the results indicated that age, dwelling status, history of mental illness, anxiety, and fear were all significant predictors of the psychological distress. Moreover, a recent survey on college students at the University of Dubai, in which 45% of respondents expressed that they did not like online learning, suggested that the pandemic had a negative psychological impact on the students’ learning [65]. Because of the lockdown, many of the students indicated that they were not used to learning effectively on their own, staying at home most of the time, when they used to study in class with their peers.

**Figure 1:**
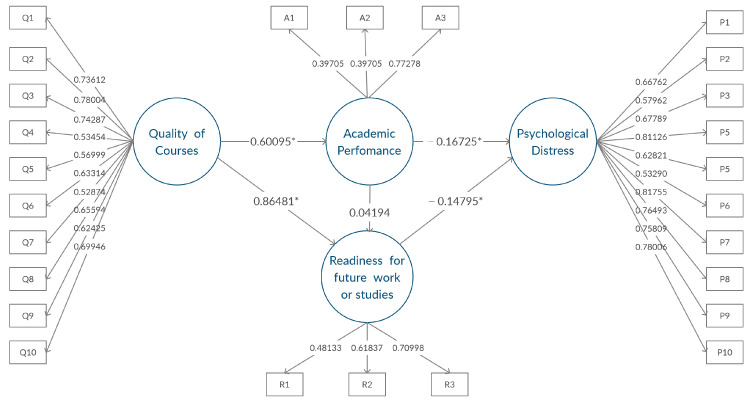
Conceptual research model, * significant relationship between latent factors.

In a nutshell, previous studies have suggested that evaluating the impact of ERT on students is a complex task with multiple dimensions of interest. Thus, it is crucial to identify specific learning outcomes influenced by the present ERT systems in order to understand and improve students’ experiences. Generally speaking, little information is available regarding the COVID-19 experiences in the UAE. More specifically, to the best of our knowledge, none of the studies conducted in the UAE has investigated the mediating role of ERT and its quality during the COVID-19 pandemic on the mental health of college students. Hence, a conceptual research model was built using the theoretical foundations given in the literature, and then re-structured to fill in the gaps in the literature and to explore the impact of ERT on college students within the UAE (see Figure 1). In this research, we analyzed the effects of perceived course quality under ERT on the specific learning constructs: academic performance and readiness for future work/studies. Additionally, we examined the impact of perceived course quality under ERT on psychological distress levels among college students in the context of these learning constructs as well as the influence of reported demographic variables on the psychological distress. The findings of this study will provide insights into the challenges that might affect college students’ mental health and will draw more attention to the factors that can put college students at higher risk of developing more psychological distress symptoms in the long run.

## 3. Methodology

The method used in this study involved three main stages: survey design, data collection, and data analysis. The data analysis stage covered measurement validation, model validation for Confirmatory Factor Analysis (CFA), Structural Equation Modeling (SEM), and univariate statistical tests.

### 3.1. Survey Design

Based on the previous literature review, a cross-sectional Google Forms designed survey was adopted. The survey included questions on demographic variables in addition to four constructs depicting the quality of courses under ERT, academic performance, readiness for future work/studies, and psychological distress. Details and descriptions of the four constructs are itemized below. Observed indicators were appraised for each of these constructs in the survey. For simplicity of coding, the questionnaire dimensions were itemized by the dimension number and represented by the first letter of the latent factor as a prefix (e.g., Q1–Q10). Students were also allowed to note any additional comments they had through one optional open-ended question at the end of the survey. Details of the questions chosen for the study are presented in Table A1 in the Appendix A. Worth noting is that the first draft of the survey was piloted with 10 students to check the clarity of the questions. As a result, some of the survey questions were revised.

i. Psychological Distress

The Kessler Psychological Distress Scale (K10) is a measure of psychological distress that encompasses a 10-item questionnaire [66]. Participants responded to these items using a 5-point time-anchored Likert scale from 0 (never) to 4 (always); the results could then be added up to obtain a final score between 0 and 40. Higher scores indicate high levels of psychological distress, whereas low scores suggest lower levels of psychological distress.

ii. Quality of Courses

Participants were asked to rate their online learning experiences on a 5-point Likert scale from 0 (never) to 4 (always), using ten items related to course delivery approach, assessment components, and interaction during the class. These items were meant to capture some of the most prevalent indicators of course quality, based on previous work. Interaction items in the survey can be categorized as student–student interaction (e.g., “There was good interaction between me and my classmates in online classes in Spring 2020”), instructor–student interaction (e.g., “I received feedback from the instructor regarding my work in Spring 2020”), or student–content interaction (e.g., “I found the online classes to be more creative than self-study in Spring 2020”) [67]. Moreover, as one of the major indicators of course quality, most items focused on self-reported student satisfaction (e.g., “I was satisfied with the live sessions in Spring 2020”).

iii. Academic Performance

To examine if ERT and its perceived quality amidst the COVID-19 pandemic had any influence on the students’ academic performance, participants were questioned about any change in their GPA after the transition to ERT in Spring 2020. For this question, one of four responses was required (i.e., “decreased”, “remained the same due to choosing Pass/No Pass”, “remained the same due to no change in my level”, or “increased”). Participants were also asked to rate their satisfaction with their assessment grades, using a 5-point frequency Likert scale from 0 (never) to 4 (always), by the items “I feel I have been graded fairly with respect to other students in my class in Spring 2020” and “I feel I deserved the final grade I received in my online courses in Spring 2020”. Although most previous studies tended to evaluate academic performance solely through GPA or test performance [49], there has been evidence of a significant positive relationship between the students’ actual grade outcomes and their perceptions regarding grade satisfaction and fairness [68].

iv. Readiness for Future Work/Studies

For the purposes of this study, we identified the scope of technical or conceptual knowledge gained from online courses as readiness for future work or studies [69]. Consequently, participants were asked to rate the adequacy of their course-obtained skills and knowledge in preparing them for the future on a 5-point Likert scale from 0 (never) to 4 (always). The three items included were “Online courses with labs or programming were effective in Spring 2020”, “During Spring 2020, I learned sufficient technical knowledge to prepare me for work”, and “During Spring 2020, I learned sufficient knowledge (from pre-requisites) to prepare me for upcoming courses”.

### 3.2. Data Collection

This study was based on the American University of Sharjah (AUS) undergraduate students from the College of Engineering; the College of Arts and Sciences; the School of Business Administration; and the College of Architecture, Art, and Design. According to the Times Higher Education (THE) World University Rankings [70], AUS is among the top three universities with the highest percentage of international students worldwide. As English is the official language of instruction at AUS, the study was conducted in English. All in all, a total of 588 undergraduate students took part in this investigation. The researchers also used a focus group of the study population who expressed their readiness to be interviewed later on in the research. This group comprised a total of 10 students: 4 students from the college of Engineering and 2 students from each of the other colleges. Additionally, four of the students were second year students while two students were selected from each of the other university levels. The students in the focus group were invited to share their attitudes, beliefs, and experiences concerning the survey questions.

As for the rest of the study participants, an approximate of 58.2% of total respondents answered the optional open-ended question at the end of the survey and provided additional remarks on ERT. The obtained insights were later considered while evaluating the data and questioning the focus group. The distributions for the years of study amongst the participants were similar, with sophomores making up for the majority of respondents (n = 179, 30.4%). Prior to the study, participants were informed that the purpose of the study was to learn about their perceptions regarding online learning. The study was completely voluntary and participants were allowed to end the survey whenever they wanted. Additionally, the study was anonymous; participants were assured of the confidentiality of their information. A summary of the demographic information of the study participants can be found in Table 2. Participants completed the online survey at their own pace. All participants received the measures in the same order. The entire survey took approximately 7–10 min to complete.

Overall, 55.1% of the respondents were females and 44.9% were males. Most respondents indicated that they were living with their parents at the time of the study (94%). As for the respondents’ previous history of distress and anxiety, about half of the total study population reported having a previous history of distress or anxiety. In addition, 41.8% of the respondents reported that their education was at risk due to financial issues caused by the COVID-19 pandemic in Spring 2020.

#### Ethical Considerations

As per AUS guidelines, an Institutional Review Board (IRB) form was submitted to the Office of the Institutional Research and an official approval was obtained to collect the necessary data. The study was carried out following all the relevant guidelines and regulations.

### 3.3. Data Analysis

The survey data was analyzed using SEM. SEM is a powerful statistical method for analyzing multivariate data with assumed mediation relations [71]. It provides a path analysis that shows direct, indirect, and total effects of one variable on another. The direct effects are those not mediated by any other variable; the indirect effects operate through at least one intervening variable, and the total effects is the sum of direct and all indirect effects [71]. Based on the dimensions found in the literature and extracted from the latent factors’ definitions, mentioned in the survey design stage, the model hypotheses were constructed as follows:

**Hypothesis** **1.** *There is a significant association between the quality of courses during ERT and the students’ readiness for future work or studies.*

**Hypothesis** **2.** *There is a significant association between the quality of courses during ERT and the students’ academic performance.*

**Hypothesis** **3.** *The students’ readiness for future work or studies has an effect on the students’ psychological distress.*

**Hypothesis** **4.** *The students’ academic performance has an effect on the students’ psychological distress.*

**Hypothesis** **5.** *There is an impact of the quality of courses during ERT on the students’ psychological distress in the context of academic performance and readiness for future work or studies.*

**Hypothesis** **6.** *There is a significant association between the students’ academic performance and the students’ readiness for future work or studies.*

**Hypothesis** **7.** *Psychological distress is influenced by some of the demographic variables.*

The last hypothesis was examined using univariate statistical tests, while H1–H6 were examined using the SEM model illustrated in Figure 1.

#### 3.3.1. Measurement Validation

Cronbach’s α and McDonald’s ω were used to evaluate the measurement reliability. Cronbach’s α is a measure of the internal consistency, used to check the correlation of each indicator with the latent factor [71]. It is generally the most widely used reliability measure across the literature. On the other hand, McDonald’s ω is computed using loadings from the factor analysis and presents a more general measure of the reliability. Moreover, McDonald’s ω is more consistent than Cronbach’s α alpha since Cronbach’s α tends to underestimate the accurate reliability if the indicators are not tau-equivalent [72]. McDonald’s ω is also an alternative that can be used for both interval and ordinal Likert scale [73]. Table 3 illustrates the main reliability and validity statistics involving Cronbach’s α and McDonald’s ω for each latent factor, in addition to the correlations with total. Cronbach’s α and McDonald’s ω for all the latent factors were higher than the acceptable level of 0.6. Indicators with low correlation with other indicators within the latent factor were removed.

In order to avoid overfitting of the data, the ratio of observations to estimated parameters has been suggested as a guidance strategy for sample size validity. A suitable ratio of observations for each estimated parameter in the model was proposed to be as high as 20 to 1, or as low as 5 to 1 [71]. In this study, CFA identifies four latent factors that are represented by a total of 26 observed indicators (see Table A1 in the Appendix A). This would result in 520 observations if the 20 to 1 ratio was used. Accordingly, we feel the sample size was sufficient, given there were 588 observations in the research.

#### 3.3.2. CFA Model

Prior to the evaluation of the structural model, CFA was conducted to validate the measurement model. Model goodness of fit indices for the CFA model demonstrated an acceptable fit. In particular, the Chi-Square was 1245.5 with 293 degrees of freedom, Standardized Root Mean Residual (SRMR) was 0.0535, Goodness of Fit Index (GFI) was 0.8510, and Bentler Comparative Fit Index was recorded at 0.8658. Moreover, all factor loadings were significant (*p*-values < 0.05) and the standardized estimates were generally higher than or close to 0.5.

#### 3.3.3. SEM Results

SEM was performed using PROC CALIS in SAS Studio software in order to investigate the relationship between the endogenous latent factors (academic performance, readiness for future work or studies, and psychological distress) and the exogenous latent factor (quality of courses) as demonstrated in Figure 1. SEM examined the impact of the manifest variables on psychological distress, academic performance, and readiness for future work or studies, simultaneously considering their correlation. In the SEM, the loadings of the manifest variables were constrained to their specific latent factors, and the model was validated using goodness of fit statistics. The estimate, *p*-value of the estimate, the confidence interval of the estimate, and the standardized loading for each of the manifest variables are shown in Table 4.

Moreover, the goodness of fit statistics for the SEM were as follows: Chi-Square was 807.5 with 289 degrees of freedom, SRMR was 0.0479, Goodness of Fit Index (GFI) was 0.9003, and Bentler Comparative Fit Index was 0.9269; the results suggested a good fit.

The results of the SEM model in terms of the significant latent factors and direct/indirect effects are displayed in Table 5. In support of H1 and H2, the results indicate that the quality of courses has a direct effect on the students’ readiness for future work or studies and on the students’ academic performance, respectively. Additionally, psychological distress appears to be significantly influenced by readiness for future work or studies and academic performance, as hypothesized in H3 and H4, respectively. However, there was no significant effect of the students’ academic performance on the students’ perceived readiness for future work or studies; hence, H6 was rejected. Eventually, SEM revealed significant evidence of an indirect impact by the quality of courses during ERT on the students’ psychological distress in the context of the students’ academic performance and their readiness for future work or studies. Hence, H5 was verified. In our model, SEM indicated that the total effects of the students’ academic performance and readiness for future work or studies on psychological distress were −0.1734 and −0.1480, respectively, with *p*-values of 0.0083 and 0.0226, respectively. The total effects of the quality of courses during ERT on the students’ readiness for future work or studies, academic performance, and psychological distress were 0.8900, 0.6009 and −0.2322, respectively, with *p*-values less than 0.0001. Nevertheless, while the results showed that the quality of courses under ERT had a significant effect on the three latent factors, the greatest effect was found to be on readiness for future work or studies.

#### 3.3.4. Multiple-Group Models

**Figure 2:**
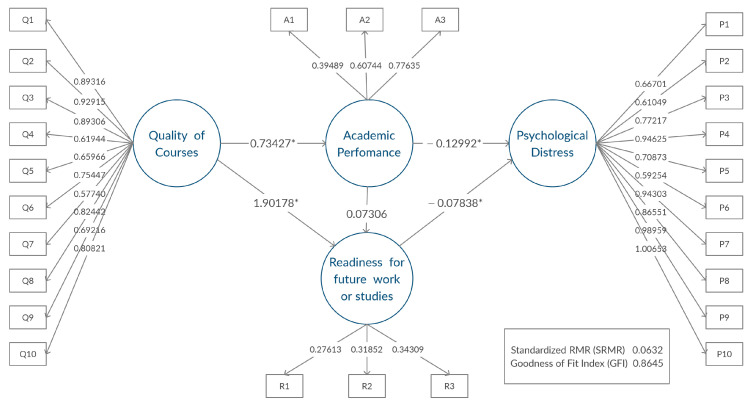
Path model for females, * significant relationship between latent factors.

**Figure 3:**
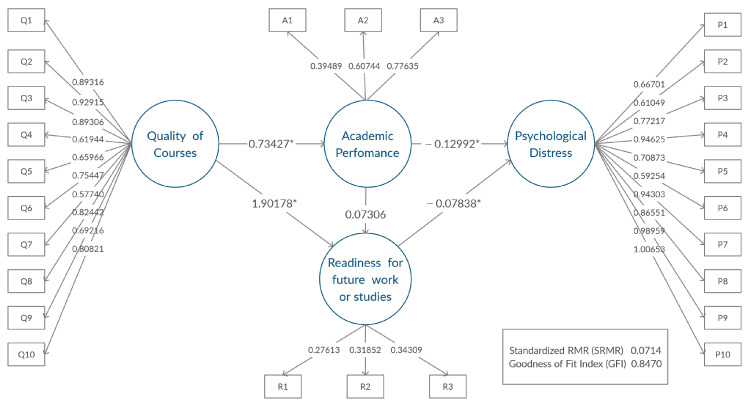
Path model for males, * significant relationship between latent factors.

In order to investigate whether the same path structure was valid, based on the Gender group, fully constrained SEM models were fitted for both the Male and Female groups. All parameters were constrained to be the same for the two models. The results showed the same path structures. In addition, all parameter estimates were found to be significant with a *p*-value less than 0.05. Figure 2 and Figure 3 describe the models obtained for the two gender groups, using the (unstandardized) estimates. The results suggested that the impact of ERT on the students’ learning outcomes and mental health behaved similarly on students of either gender. The goodness of fit statistics for the multiple-group SEM models were as follows: Chi-Square with a value of 1273.7 and 614 degrees of freedom, SRMR was 0.0670, Goodness of Fit Index (GFI) was 0.8566, and Bentler Comparative Fit Index was 0.9085. The values indicated a good fit.

#### 3.3.5. Univariate Statistical Tests

**Figure 4:**
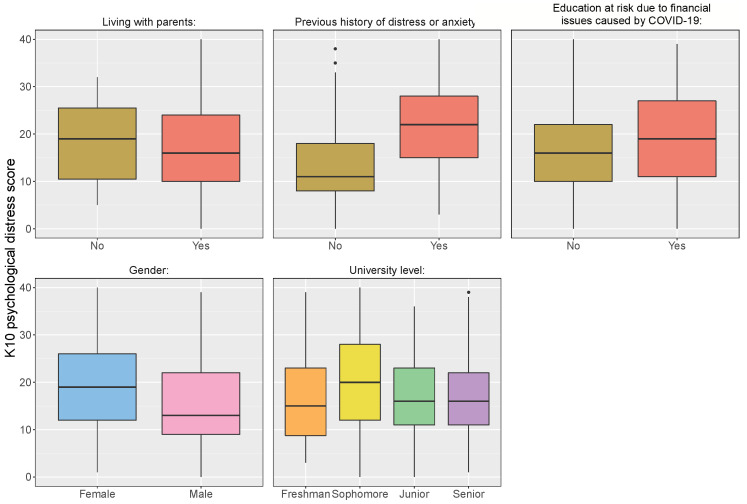
Psychological distress of demographic variables.

The demographic variables reported in the survey were also investigated with respect to their effects on the students’ psychological distress levels. As described in Section 3.1, the Kessler (K10) Psychological Distress items were summed up to obtain a final score between 0 and 40; these scores were indicative of the level of psychological distress. As these scores came from Likert Scale data, a non-parametric analogue of ANOVA, Kruskal–Wallis (K-W) test, was used for comparing the medians of the distress scores (summed scores) for each group within the year of study demographic variable. At 5% significance level, the K-W test showed evidence of significant differences between the various years of study with a *p*-value of 0.0012. Post hoc pairwise comparisons between the groups suggested that the significant differences between medians come from comparing sophomores with senior (*p*-value = 0.0113) and sophomores with freshmen (*p*-value = 0.0026); the p-values were adjusted to control type I error [74]. Moreover, the median difference in reference to sophomores was at least 4 units (see Figure 4).

In terms of gender, a Mann–Whitney U test presented strong evidence of differences between the median of psychological distress scores in males versus females (*p*-value < 0.0001). That is, the median of psychological distress scores in females was higher than in males by about 6 units. Similarly, a significant difference was observed with respect to having a previous history of anxiety or distress (*p*-value < 0.0001). The median of psychological distress scores for students with no previous history of anxiety or distress was lower by 10 units than those with a previous history. Moreover, it was found that there is a significant difference between the median of psychological distress scores for the students whose education was at risk due to financial issues caused by COVID-19 (*p*-value = 0.0002). On the contrary, there was no significant difference between the median of psychological distress scores for college students who were living with their parents versus college students who were living away from their parents (*p*-value = 0.6314). In conclusion, the findings supported the hypothesis stated in H7.

## 4. Discussion

### 4.1. SEM Results Discussion

In this study, several significant factors impacted by the transition to ERT on college students were investigated. The conceptual research model was built (see Figure 1) in order to illustrate the influence of the quality of courses during ERT on academic performance, psychological distress, and readiness for future work or studies. Most notably, SEM revealed that the quality of courses during ERT had a significant impact on the students’ perceived readiness for future work or studies (H1), and their academic performance (H2). Possibly due to the abrupt shift to ERT, instructors might have opted to focus on teaching conceptual knowledge at the expense of procedural knowledge. However, while conceptual knowledge is important to succeed in today’s labor market, it is essential for graduates to acquire procedural knowledge, with a focus on skills that are transferable across various domains and contexts in their careers [75]. This will also equip students with what is known in academia as long life learning skills [69]. Particularly, these transferable and lifelong skills are the the main qualifications that satisfy the requirements needed to meet the criteria for today’s job market [75].

Therefore, students who did not feel that they acquired sufficient background in preparation for the workforce or future studies might have tended to be more worried about finding a job and were at a higher risk of developing psychological distress symptoms. This conclusion validated (H3). It also supported (H5), which stated that the quality of courses has an indirect effect on the students’ psychological distress when the perceived readiness served as a mediating factor. Thus, university administrations should collaborate with instructors who teach courses that include technical lab work to devise the most suitable and effective methods for allowing the students to obtain this technical knowledge and to enhance the quality of courses taught during ERT. Some possible suggestions for improving the learning experience in this regard include the implementation of open-ended course projects coupled with short synchronous one-on-one meetings with students [76,77].

In line with the previous implications, the analysis supported H4, which indicated that academic performance has an effect on the psychological distress of college students. Students who were in the focus group reported that they tended to worry about achieving a minimum GPA in order to graduate on time or maintain a scholarship or financial aid. Various universities, including AUS, gave students the option to choose Pass/No Pass (P/NP) in Spring 2020 for any of their courses [78]. This approach allowed students to remove unfavorable grades from their cumulative GPA during the COVID-19 pandemic. However, many students from the focus group remained concerned that their grades might be interpreted negatively if they opted for the P/NP option. These students expected the university to evaluate their probation status, applications for scholarships, or financial aids on the basis of their actual GPA before choosing the P/NP option. Additionally, survey and focus group respondents indicated that they were worried about delaying their graduation if they failed courses or failed to obtain financial aid. Some students from the focus group mentioned that they felt they were not graded fairly when comparing their grade to their peers and the class average. It is possible that these concerns contributed to the negative association found between academic performance and psychological distress. Additionally, since the assessment components are crucial parts of the quality of courses, these concerns might also explain the negative indirect effect of the quality of courses on the students’ psychological distress when academic performance served as a mediating factor (H5). These findings were consistent with previous literature reporting that there was a negative association between academic performance and psychological distress [79].

Conversely, survey and focus group respondents reported that internet access was not a challenge for them during ERT. This might be attributed to the remarkable support efforts the UAE government has been exerting in providing all students with access to the internet since the outbreak of the COVID-19 pandemic. For instance, on 8 March 2020, the Telecommunications Regulatory Authority in the UAE announced that free internet access would be provided to students in order to help them attend classes through the various e-learning platforms [80]. Nevertheless, some students from the focus group indicated that they, sometimes, faced technical problems while conducting exams, which made them stressed and affected their performance.

As for the effect of academic performance on the students’ readiness for future work or studies, little evidence was found. This might be the case because academic performance does not actually measure the technical or the theoretical knowledge obtained from the course [75]. Alternatively, it is possible that the online assessment tools might not be suitable indicators of the students’ knowledge in a course. In the focus group results, some students pointed out that they were nervous or anxious during online assessments and that their exam performance had been accordingly compromised. In such a case, the online assessment results might not be fully reflective of the students’ conceptual or technical understanding of the subject. Therefore, H6 was rejected. Moreover, since the students may achieve lower grades and not show satisfying academic performance, this could associate with increasing levels of psychological distress, which aligns with H4.

To aid in the design and construction of effective online assessments, we advise that course instructors maintain a balance between summative and formative assessments [81]. Summative assessments, which have higher stakes, serve the aim of evaluating students’ success at or around the end of a course of study. Formative assessments, on the other hand, are commonly performed by teachers throughout a course to measure student learning as it occurs. With the transition to ERT, frequent and varied low-stakes formative evaluations, such as quizzes, allow students to become acquainted with online assessment methods and improve their engagement [82]. In addition, allowing more than one attempt in formative assessments, diversifying assessment techniques, and ensuring high-quality and timely feedback are all applicable ways of facilitating student learning during ERT [83,84,85].

From the above, ERT could have considerable influences on the students’ mental health and their careers on the long run. While providing social support and targeted interventions with respect to the mental health of college students is recommended, the results of the present study show that course instructors should play a more guiding and supporting role for the students and work on improving online course delivery. Given the psychological effects of ERT, conveying care and implementing course flexibility by faculty members are all suggested to be crucial components in promoting the students’ well-being and achievement [86,87]. This could be achieved through allowing the students greater liberty in meeting course demands, alleviating assessment time constraints, reacting to student concerns on a regular basis, and providing more accessible teaching materials [87,88,89]. On that account, our findings also recommend that specialized training be provided for all faculty members. Per previous work, faculty who were trained even after the shift to ERT were the most likely to report a positive educational experience, demonstrating that training can be effective at any time [90].

### 4.2. Multiple-Group Models Discussion

It is worth mentioning here that performing multiple-group SEM for a gender group did not reveal any significant differences in the path diagrams and the relationships between the latent variables. This indicates that our discussed impact of ERT has equivalent properties across both male and female students.

### 4.3. Univariate Test Discussion

Overall, in support of H7, most of the demographic variables recorded were found to have a significant impact on psychological distress. In terms of gender, results indicated that there was statistical evidence of differences between the psychological distress levels between male and female students. It was found that female college students tended to have a higher median K10 score in comparison to males. This is in line with previous literature that highlights women as being more prone to distress measures [5]. In addition, having a previous history of anxiety appeared to be more positively associated with having psychological distress during ERT. Although this finding conflicted with a recent longitudinal study in which students without pre-existing mental health concerns were likelier to exhibit declining mental health during COVID-19 [91], the results were in line with prior findings on mental health in the UAE [63,64].

Furthermore, performing Kruskal–Wallis analysis to compare the median K10 psychological distress scores among different years of study revealed critical results. In contrast to other years of study, it was indicated that sophomores tended to have higher median K10 scores, whereas little evidence revealed differences between freshmen, juniors, and seniors. These results might be attributed to the fact that sophomores (i.e., second year students) have already experienced the physical classroom environment before switching to ERT in the midst of their major core courses. According to the AUS catalog [92], major core courses start in the second year for almost all of the university study disciplines. Sophomores have experienced going to the university for their first academic year, knowing new people, creating a new social experience, and being independent from their families. Accordingly, they might be more vulnerable to the transition to ERT amidst the COVID-19 pandemic in terms of losing these experiences.

In addition, a higher median of the K10 psychological distress scores was found significant for college students whose education was at risk due to financial issues caused by COVID-19. The COVID-19 shutdown has had a significant influence on the worldwide economy, causing some to fear a global recession [7]. According to a recent study, many people experienced increased financial anguish as a result of the uncertainty of the economic environment caused by the COVID-19 pandemic [7]. Particularly, students from the focus group indicated that they were afraid of leaving the country at anytime or losing an academic semester and graduating late due to being unable to maintain their scholarships or to pay their college fees. Hence, to overcome this adversity, the National Health System should take action to provide financial assistance and to foster accessible treatment options for mental health problems [7].

On a final note, living with parents was found to be insignificant in affecting the psychological distress scores. Previous literature has indicated that parents’ psychological distress, which aligns with stressing over their children’s behavioral or emotional problems, declines in line with increased child age [93]. Another study showed that parents experience a transition in the way they deal with their children as they move to college [94]. It is also possible that survey respondents who lived with guardians other than their parents or had strong social support contributed to the insignificance of the test result.

## 5. Conclusions, Limitations, and Recommendations

This study aimed to provide a comprehensive overview of the most significant learning constructs impacted by the transition to ERT in college students within the UAE and its subsequent influence on their mental health. It was found that the quality of courses had a significant effect on the students’ academic performance and on their perceived readiness for future work or studies. Due to the new system of online teaching during ERT, more focus might have been attributed to conceptual knowledge at the cost of acquired technical experience. College students who felt that they lacked important technical knowledge were more likely to develop psychological distress symptoms, as psychological distress was negatively associated with the students’ academic performance and their readiness for future work or studies. SEM analysis also revealed an indirect significant effect of the quality of courses during ERT on the students’ psychological distress. On the other hand, it was found that there was no significant association between the students’ academic performance and their readiness for future work or studies. This result was supported by the fact that online assessments could not be fully representative of the students’ conceptual or technical knowledge of the courses as understood from the students who were in the focus group. In addition, the results suggested higher distress scores among female students, sophomores, those with a previous history of distress/anxiety, and students whose education had been at risk due to financial issues caused by COVID-19. Nevertheless, it was found that there was no significant difference on psychological distress between the students who were living with their parents and the students who were living away from their parents. It was also observed that there was no significant differences between males and females in the the multiple-group SEM, which indicated that influence of the sudden shift to ERT affects female and male college students in similar ways.

The results also revealed that effective and robust social support is necessary for college students during public health emergencies, especially considering that the psychological impacts of COVID-19 are expected to linger long after the pandemic has peaked [95,96]. Therefore, our findings are meant to help governments, policymakers, universities’ administrations, and instructors improve the quality of courses during the COVID-19 pandemic and provide additional support to students. It is noteworthy to mention that this study was based on undergraduate students at AUS. Thus, a generalization of the results to all undergraduate students in the UAE requires more validation. Moreover, this study did not examine other factors such as students’ study habits and how they affect academic performance and readiness for future work or studies. Since the successful implementation of e-learning systems is often influenced by the students’ attitudes towards them and on how they perceive it as well [55], future researchers may consider investigating any effects between these factors. Moreover, this study was focused on college students only. Thus, further research may probe into instructors’ perceptions on the impacts of ERT on students and on the whole ERT process. Another construct for further explorations could be examining the effect of the abrupt transition from face-to-face to ERT on the relationship between instructors and learners. Finally, there are other seismographic variables that can be investigated in future studies to explore their effects on college students’ mental health, such as having a family member who has been diagnosed as positive for COVID-19 or losing a family member who had suffered from the symptoms of COVID-19.

## Figures and Tables

**Table 1:** Dimensions of assessing the impact of ERT.

Dimension	Najmul and Yukun [46]	Kapasia et al. [54]	Cao et al. [55]	Almaiah et al. [56]	Aucejo et al. [27]	Aristovnik et al. [57]	Al-Tammemi et al. [58]	Aguilera-Hermida [37]
Quality of ERT								
1-Finding the course clumsy	X			X		X		
3-Task performance and engagement	X							X
4-Performance feedback	X			X				
5-Instructor Support	X	X				X		
6-Online assessment	X							
7-Interaction with peer students					X			X
Learning conditions								
1-Study Habits		X			X			X
2-Learning environment		X						X
3-Motivation for study						X	X	X
Technological ease								
1-Access to the internet/technology	X	X	X	X		X		X
2-Availability of technical assistance				X				X
2-Ease of online procedures	X			X				X
Student fears								
1-Worry about academic delays	X	X	X					
2-Worry about career development						X		
3-Worry about public examinations	X				X			
4-Worry about academic year decision	X							
5-Worry about future studies						X		
6-Fear of losing academic year					X			
Financial issues								
1-Steady family income		X	X	X	X			
2-E-Learning costs	X							
Mental health								
1-Previous history of mental health challenges							X	
2-Feelings of anxiety or depression	X	X	X			X	X	X
3-GAD7 (Anxiety levels)			X					
4-K10 (Kessler Psychological Distress)	X						X	

X shows that this dimension was mentioned in the indicated reference.

**Table 2:** Demographic information of the participants.

Demographic Information	Response	Frequency (n)	Percentage (%)
**Gender**	1. Female	324	55.10%
	2. Male	264	44.90%
**Year of study**	1. Freshman	160	27.20%
	2. Sophomore	179	30.40%
	3. Junior	94	16.00%
	4. Senior	155	26.40%
**Living with Parents**	1. No	35	5.90%
	2. Yes	553	94.0%
**Education at risk due to financial** **issues caused by COVID-19**	1. No	342	58.20%
	2. Yes	246	41.80%
**Previous history of distress or anxiety**	1. No	283	48.10%
	2. Yes	305	51.90%

**Table 3:** Construct reliability.

Factors	Correlation with Total	Cronbach’s Alpha	McDonald’s Omega
Psychological Distress		0.913	0.914
P1	0.643		
P2	0.601		
P3	0.695		
P4	0.751		
P5	0.621		
P6	0.542		
P7	0.780		
P8	0.737		
P9	0.713		
P10	0.738		
Academic Performance		0.622	0.678
A1	0.317		
A2	0.435		
A3	0.557		
Quality of Courses		0.882	0.883
Q1	0.702		
Q2	0.739		
Q3	0.684		
Q4	0.515		
Q5	0.546		
Q6	0.596		
Q7	0.493		
Q8	0.607		
Q9	0.576		
Q10	0.642		
Readiness for Future Work/Studies		0.640	0.659
R1	0.420		
R2	0.530		
R3	0.410		

**Table 4:** Estimates of the ERT measurement constructs.

Path	Estimate	*p*-Value	95% Confidence Interval		Standardized Estimate
**P => P1**	0.68726	<0.0001	0.60996	0.76455	0.66762
**P => P2**	0.63511	<0.0001	0.54977	0.72045	0.57962
**P => P3**	0.79095	<0.0001	0.70369	0.87821	0.67789
**P => P4**	0.96220	<0.0001	0.87927	1.04512	0.81126
**P => P5**	0.72062	<0.0001	0.63300	0.80824	0.62821
**P => P6**	0.60237	<0.0001	0.51296	0.69178	0.5329
**P => P7**	0.94348	<0.0001	0.86286	1.02409	0.81755
**P => P8**	0.87302	<0.0001	0.79064	0.95540	0.76493
**P => P9**	1.00049	<0.0001	0.90509	1.09588	0.75809
**P => P10**	1.01884	<0.0001	0.92564	1.11205	0.78006
**A => A1**	0.39055	<0.0001	0.29959	0.48151	0.39705
**A => A2**	0.60584	<0.0001	0.52054	0.69113	0.67014
**A => A3**	0.76181	<0.0001	0.65890	0.86471	0.77278
**Q => Q1**	0.89628	<0.0001	0.80809	0.98446	0.73612
**Q => Q2**	0.92582	<0.0001	0.84214	1.00950	0.78004
**Q => Q3**	0.89197	<0.0001	0.80582	0.97812	0.74287
**Q => Q4**	0.61865	<0.0001	0.52779	0.70950	0.53454
**Q => Q5**	0.65395	<0.0001	0.56498	0.74291	0.56999
**Q=> Q6**	0.74931	<0.0001	0.65980	0.83883	0.63314
**Q => Q7**	0.57746	<0.0001	0.49155	0.66336	0.52874
**Q => Q8**	0.82296	<0.0001	0.72899	0.91692	0.65594
**Q => Q9**	0.68880	<0.0001	0.60502	0.77257	0.62425
**Q => Q10**	0.80383	<0.0001	0.71950	0.88815	0.69946
**R => R1**	0.27442	<0.0001	0.19426	0.35458	0.48133
**R => R2**	0.31450	<0.0001	0.22922	0.39978	0.61838
**R => R3**	0.34260	<0.0001	0.25051	0.43469	0.70998

**Table 5:** SEM path analysis.

Effect	Path	Estimate	SE	*p*-Value	Std. Estimate
Direct Effects	Quality of Courses =>Academic Performance	0.7519	0.0756	<0.0001	0.6009
	Quality of Courses =>Readiness for Future Work/Studies	1.9019	0.2960	<0.0001	0.8648
	Academic Performance =>Psychological Distress	−0.1392	0.0570	0.0146	−0.1672
	Readiness for Future Work/Studies =>Psychological Distress	−0.0701	0.0319	0.0281	−0.1480
	Academic Performance =>Readiness for Future Work/Studies	0.0737	0.1075	0.4929	0.0419
Indirect Effects	Quality of Courses =>Psychological Distress	−0.2418	0.0455	<0.0001	−0.2322

## Data Availability

The data presented in this study is available on request from the corresponding author. The data is not publicly available due to confidentiality reasons.

## Appendix A

**Table A1:** Construct details.

Item	Item Details
*Quality of Courses*	
Q1	I was satisfied with the live sessions in Spring 2020.
Q2	I was satisfied with the material delivery, lecture notes, and practice problems in Spring 2020.
Q3	I was satisfied with the assessment components: assignments, quizzes, exams, or instructor flexibility in Spring 2020.
Q4	I found the online classes to be well-organized in Spring 2020.
Q5	I found the online classes to be more creative than self-study in Spring 2020.
Q6	The quality of the online courses in Spring 2020 was the same as face-to-face learning.
Q7	There was good interaction between me and my classmates in online classes in Spring 2020.
Q8	I was satisfied with the instructors’ office hours in Spring 2020.
Q9	I received feedback from the instructor regarding my work in Spring 2020.
Q10	I was satisfied with the communication tools in Spring 2020: e-mails, discussion boards, Whatsapp, etc.
*Academic Performance*	
A1	After Spring 2020, my GPA has (increased/decreased/remained the same).
A2	I feel I have been graded fairly with respect to other students in my class in Spring 2020.
A3	I feel I deserved the final grade I received in my online courses in Spring 2020.
*Psychological Distress*	
P1	I often felt tired for no good reason.
P2	I often felt nervous.
P3	I often felt so nervous that nothing could calm me down.
P4	I often felt hopeless.
P5	I often felt restless or fidgety.
P6	I often felt so restless that I could not sit alone.
P7	I often felt depressed.
P8	I often felt so sad that nothing could cheer me up.
P9	I often felt worthless.
P10	I often felt that everything is worthless.
*Readiness for Future Work/Studies*	
R1	Online courses with labs or programming were effective in Spring 2020.
R2	During Spring 2020, I learned sufficient technical knowledge to prepare me for work.
R3	During Spring 2020, I learned sufficient knowledge (from pre-requisites) to prepare me for upcoming courses.
